# Case report: Colonic actinomycosis – A rare cause of a locally advanced colonic tumour

**DOI:** 10.1016/j.ijscr.2023.107957

**Published:** 2023-03-03

**Authors:** Matthew-Anthony Lyew, Conrad Morris, Kevan Smith, Memory Stennett

**Affiliations:** a Department of General Surgery, Kingston Public Hospital, Kingston, Jamaica; b Department of Surgery, Radiology, Anaesthesia and Intensive Care, University Hospital of the West Indies, Mona, Jamaica; c Department of Pathology, National Public Health Laboratory, Kingston, Jamaica

**Keywords:** Actinomycosis, Actinomyces, Colon cancer mimic, Colonic tumour, Locally advanced, Immunocompromised

## Abstract

**Introduction and importance:**

Colon cancer is a common malignancy and is often encountered initially as locally advanced disease. However, there are many benign clinical entities that may masquerade as complicated colonic malignancy. Abdominal actinomycosis is one such rare mimic.

**Case presentation:**

A 48-year-old female presented with a progressively enlarging abdominal mass with skin involvement and clinical features of partial large bowel obstruction. Computed tomography (CT) revealed a mid-transverse colonic lesion at the centre of an inflammatory phlegmon. At laparotomy, the mass was found to be adherent to the anterior abdominal wall, gastrocolic omentum, and loops of jejunum. En block resection was performed with primary anastomosis. Final histology showed no evidence of malignancy, but mural abscesses containing pathognomonic sulphur granules and actinomyces species.

**Clinical discussion:**

Abdominal actinomycosis, particularly of the colon, is rare and exceptionally so in immunocompetent patients. However, the clinical and radiographic presentation often mimics more common conditions such as colon cancer. Accordingly, surgical resection is typically radical to clear margins, and diagnosis is made only on final histopathology.

**Conclusion:**

Colonic actinomycosis is an uncommon infection but the diagnosis should be considered particularly in colonic masses with anterior abdominal wall involvement. Oncologic resection remains the mainstay of treatment and the diagnosis commonly made retrospectively given the rarity of the condition.

## Highlights

• Most locally advanced colonic masses are treated as presumed adenocarcinoma.
• Cross-sectional imaging is usually performed and may reveal features of luminal masses or invasion.
• Multi-visceral en block resection is then typically performed to clear margins.
• Uncommonly, final histology may show rare benign findings such colonic actinomycosis.

## 1 Introduction

Colonic adenocarcinoma is the most common intra-abdominal malignancy and ranks third overall worldwide and within Jamaica at an incidence of 10.6 per 100,000 population [1]. Furthermore, up to 20–30 % of these patients will be either metastatic or locally advanced (American Joint Committee on Cancer 8th Edition T4b) at the time of presentation [2], [3]. Thus, patients who present with palpable intra-abdominal masses and typical large bowel gastrointestinal symptoms are often clinically diagnosed with a colonic malignancy until proven otherwise.

Such patients are then investigated with cross-sectional imaging whereby findings of a colonic mass lesion, mesenteric lymphadenopathy and metastatic deposits within the abdomen or chest further corroborate the presumed diagnosis of colon cancer. However, whether from biopsy or surgical resection, final histopathology remains the gold standard for diagnosis of colonic adenocarcinoma. Interestingly, it is not uncommon for this histology to show other results such as inflammatory bowel disease or non-adenomatous neoplasms. In the absence of specific patient risk factors or strongly atypical history other differential diagnoses such as infectious masses are typically considered esoteric.

Herein, the authors present a case of one such rare cause of a locally advanced colonic tumour, actinomycosis. This manuscript was prepared in accordance with the SCARE 2020 guidelines [4].

## 2 Presentation of case

A 48-year-old female with no known chronic illnesses presented to the emergency department with a 1-month history of an epigastric mass. The mass had been progressively increasing in size and was associated with intermittent abdominal pain, early satiety and new onset constipation. However, the patient continued to pass flatus and reported no vomiting, spurious diarrhoea or haematochezia. There was no personal or family history of malignancy, no constitutional symptoms and no history of prior colonic or upper gastrointestinal evaluation, with no previous surgeries. The patient had now presented acutely due to a 2-day history of worsening and now constant abdominal pain.

Physical examination revealed an afebrile well-nourished middle-aged woman in mild painful distress. Significantly, the abdomen was obese and tender over an obvious 10 cm × 10 cm firm immobile epigastric mass with associated guarding and rebound. The mass was fixed to overlying skin which itself was indurated, erythematous and oedematous. The remainder of abdominal and other systemic examinations was unremarkable. A clinical diagnosis of locally advanced and complicated colon cancer was made and the patient investigated with CT-abdomen after commencement of empiric antibiotics.

**Fig. 1:**
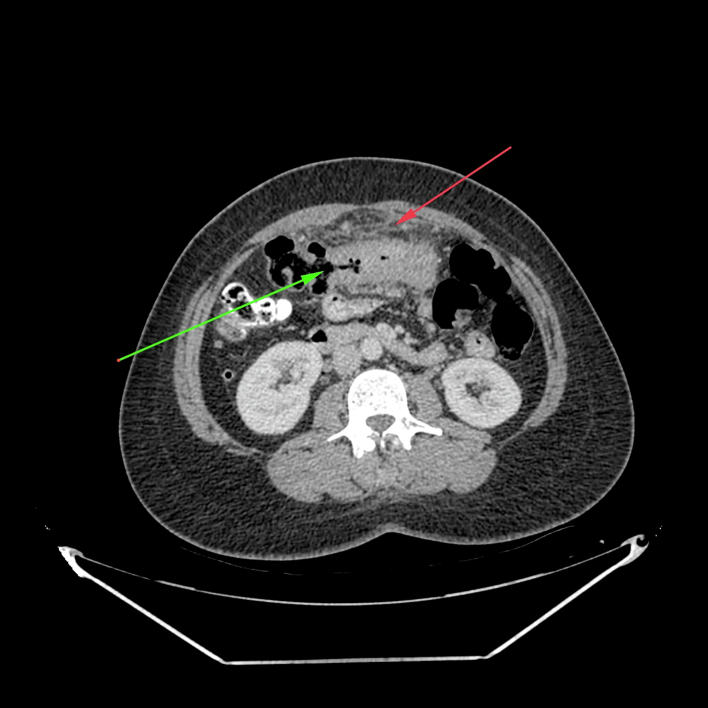
Axial CT view showing luminal mass lesion demonstrating shouldering “Apple core” deformity (green) with surrounding inflammatory infiltration into anterior abdominal wall (red). (For interpretation of the references to colour in this figure legend, the reader is referred to the web version of this article.)

**Fig. 2:**
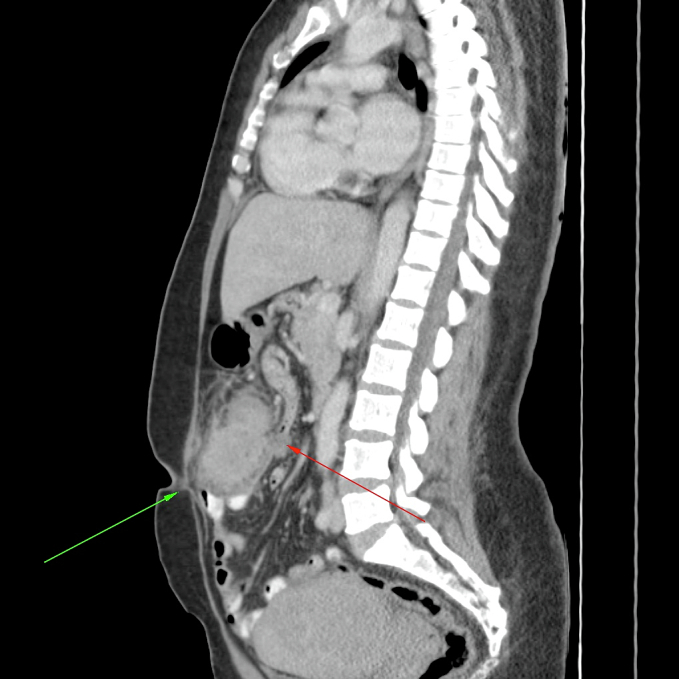
Sagittal CT view showing invasion of the mass into the peri-umbilical skin (green) and attachment to loops of small bowel posteriorly (red). (For interpretation of the references to colour in this figure legend, the reader is referred to the web version of this article.)

The CT-scan demonstrated an 8 cm long “*Apple Core*” lesion to the mid-transverse colon which extended into the greater omentum and anterior abdominal wall forming a complex 7.7 cm × 4.8 cm × 4.5 cm inflammatory mass with an associated solitary enlarged mesenteric lymph node (Fig. 1). Furthermore, the phlegmon was intimately related to loops of small bowel at its inferior border with surrounding fat stranding (Fig. 2). Additionally, bilateral solitary sub-centimetre pulmonary and pleural nodules were also noted. There was no evidence of bowel obstruction, intra-abdominal metastases or collections. The diagnosis was changed to perforated colon cancer and the patient prepared for laparotomy due to localised peritonitis and concerns of intra-abdominal sepsis.

**Fig. 3:**
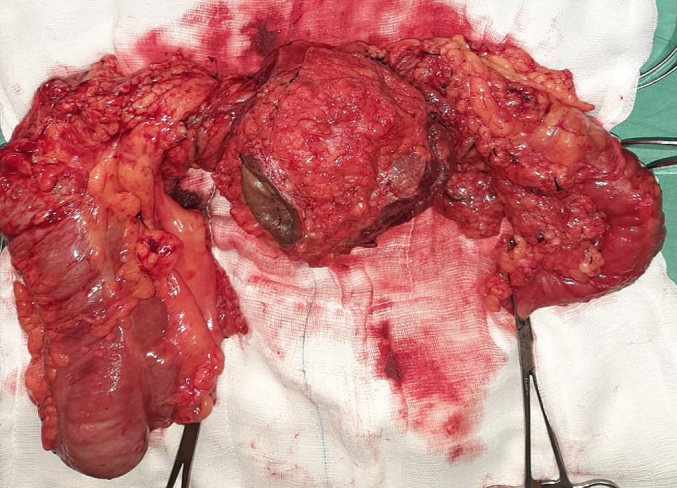
Anterior view of en bloc resection specimen with umbilicus in situ.

**Fig. 4:**
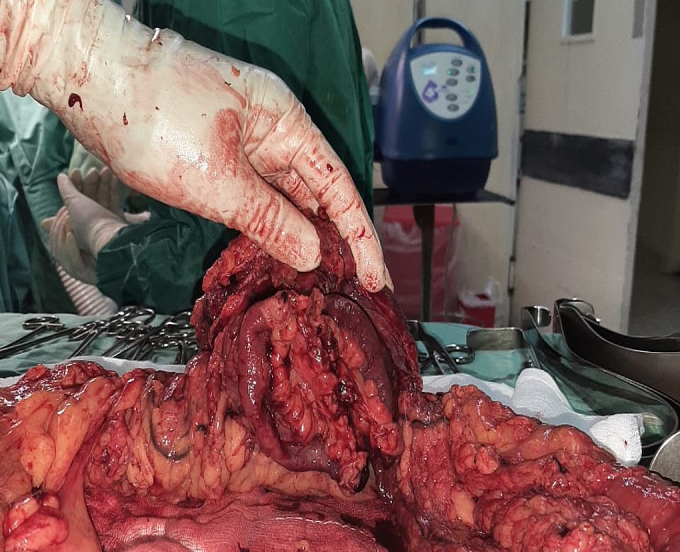
Adherent segments of small bowel inferiorly.

At surgery, a firm mid-transverse colon mass was found adherent to the anterior abdominal wall and skin as described on imaging but also posteriorly to 2 loops of jejunum approximately 260 cm and 440 cm from the duodenal-jejunal flexure. The remainder of the abdominal visceral and peritoneum were grossly normal. An en bloc resection (extended right hemicolectomy, anterior abdominal wall resection and segmental small bowel resections) was performed in an uneventful procedure (Fig. 3, Fig. 4). The post-operative convalescence was unremarkable and the patient discharged for review after completion of a 1-week course of ceftriaxone.

**Fig. 5:**
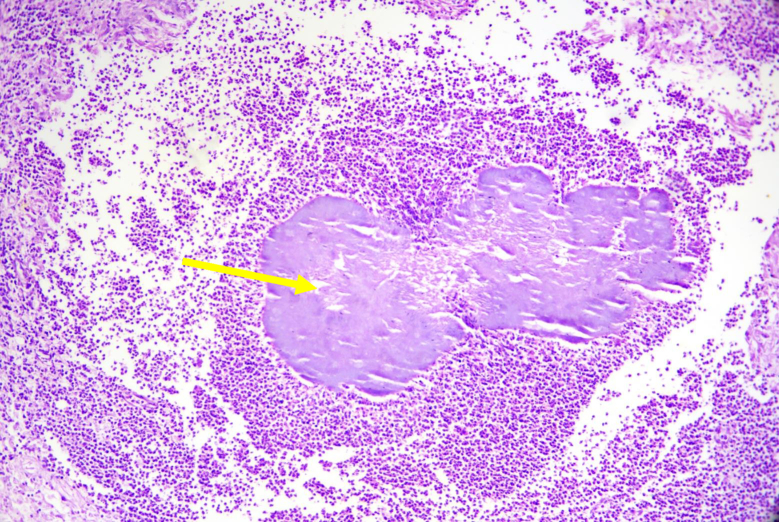
Sulphur granules with actinomyces (arrow) surrounded by significant polymorph infiltrate.

**Fig. 6:**
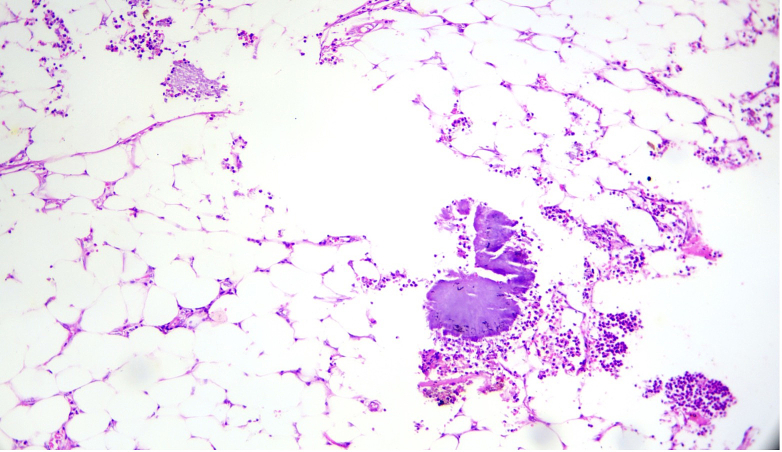
Actinomyces colonies seen in peri-colonic fat, likely appendices epiploicae or omentum.

Surprisingly, final histopathology found no evidence of malignancy but instead showed an inflammatory mass comprising of multiple transmural abscesses of the involved small and large bowel. Interestingly, these abscesses contained the characteristic sulphur granules with embedded bacterial colonies reminiscent of actinomyces (Fig. 5, Fig. 6). There was also associated dense fibrous tissue that was diffusely infiltrated with chronic inflammatory cells. A total of 15 lymph nodes were harvested, all of which showed reactive changes. A final diagnosis of colonic actinomycosis was made and the patient was seen up to 6-weeks post operatively doing well. Additional investigations done for an immunocompromised state all returned negative and a follow-up plan was made for screening of the remnant colon with flexible sigmoidoscopy in 6-months.

## 3 Discussion

Actinomycosis is a rare chronic suppurative infection caused by the commensal filamentous gram-positive anaerobic bacteria of the Actinomycetaceae family, most commonly Actinomyces israellii [5]. Of the 4 described clinical subtypes (cervico-facial, thoracic, abdominal and pelvic), abdominal actinomycosis accounts for only 20 % of cases [6]. Of these, the colon is the primary source only 14 % of the time with the ileo-caecal region most commonly involved (up to 65 %) [6], [7], [8]. Moreover, in the absence of other risk factors such as male sex, indwelling intra-uterine contraceptive device, immunocompromised state or prior surgery, this case is particularly atypical [6].

Clinicians need to remain cognisant of alternative diagnoses to malignancy and have a heightened index of suspicion in patients with large colonic masses with multi-organ involvement. Confirmation of a benign diagnosis, such as actinomycosis, may reduce surgical morbidity by affording less radical resection when possible. Interestingly, non-operative therapy with long course antibiotics (6–12-months) is well described for actinomycosis but has limited utility when unable to effectively rule out malignancy [9]. Unfortunately, the presence of large abscesses not amenable to percutaneous drainage or soft tissue necrosis, for which the condition is notorious, often necessitate operative intervention [6]. As performed in the index case, en bloc resection is often necessary given a presumptive pre-operative diagnosis of locally advanced cancer. Following surgery, in contrast to the aforementioned conservative treatment, there is far less consensus on the duration of antibiotics [6], [9]. Proposed regimes vary widely not only in duration but also in choice of agent, dosage, pelvic versus abdominal involvement, as well as clinical response [9]. There is no role for chemotherapy in the treatment of this infectious condition. We opted for a short course of antibiotics with close clinical follow-up as our patient had no known risk factors and had been doing well off antibiotic therapy while awaiting final histopathology.

The clinical and radiographic features of this presentation, as is commonplace with colonic actinomycosis, strongly overlapped with complicated malignancy. However, closer analysis of the case highlights evidence that could have heightened suspicions of an ulterior diagnosis. For example, the relatively young age of the patient and short history in conjunction with the rapid progression and locally advanced nature of the lesion. Particularly the anterior abdominal wall involvement and abscess formation, that is a well-documented characteristic of actinomyces infection, is rarely seen in colon cancer [5], [10]. Moreover, the presence of presumed isolated thoracic “metastases” seen on CT occurs in less than 10 % of patients with colorectal malignancy and at that size are highly non-specific [11], [12]. Despite these findings, notwithstanding the distinctive yellow sulphur-stained cutaneous discharge, pre-operative diagnosis occurs less than 10 % of the time [6]. When suspected, percutaneous sampling may be done for both culture and microscopy but, actinomyces has notoriously fastidious growth conditions and, the pathognomonic sulphur granules are only seen in approximately 50 % of cases [6], [13].

Further delineation from malignancy maybe done by careful evaluation of cross-sectional imaging. Firstly, due to the size of the bacterium it does not easily spread via lymphatics [14]. Therefore, although the infiltrative nature of these infections closely mimic neoplasms on CT, the paucity of lymphadenopathy despite the significant “tumour” burden is characteristic of actinomycosis [14], [15]. Notably, in the index case only 15 nodes were retrieved despite the extent of en block resection. Additionally, even though involvement of the omentum and adjacent peritoneal surfaces being typical, in contrast to malignancy, distant deposits and ascites are seldom seen [15], [16].

Finally, in the non-emergent setting, colonoscopy also aids in differentiation from neoplastic disease. Most commonly a luminal mass is not appreciated and mucosal changes, present in only 50 % of cases, are usually minimal [16]. The combination of such underwhelming endoscopic findings and an extensive extramural inflammatory mass on imaging is probably the most distinguishing feature between actinomycosis and adenocarcinoma [17]. Nevertheless, the majority of patients progress to surgery with the presumed diagnosis of complicating neoplasm, oncologic resection performed and diagnosis made in retrospect after final histology.

## 4 Conclusion

Actinomycosis is a rare cause of a locally advanced colonic tumour with clinical and radiographic features that closely resemble malignancy. Pre-operative diagnosis is ideal but difficult. It relies heavily on a high-index of suspicion in conjunction with cross-sectional imaging, percutaneous biopsy and when possible colonoscopy. Accordingly, given inability to rule out malignancy, surgery is often indicated and the diagnosis made in retrospect. Response to surgery may be variable and the ideal adjuvant antibiotic regime is likely patient specific and hinges on good clinical judgement.

## Consent

Written informed consent was obtained from the patient for publication of this case report and accompanying images. A copy of the written consent is available for review by the Editor-in-Chief of this journal on request.

## Ethical approval

This anonymised case report is exempt from ethical approval.

## Funding

This research did not receive any specific grant from funding agencies in the public, commercial or not-for-profit sectors.

## Guarantor

Matthew-Anthony Lyew.

## Research registration number

Not applicable.

## CRediT authorship contribution statement

**Matthew-Anthony Lyew:** Conceptualization, Visualization, Writing – original draft. **Conrad Morris:** Writing – review & editing. **Kevan Smith:** Writing – review & editing. **Memory Stennett:** Resources.

## Declaration of competing interest

None to declare.
